# The Prevalence of Anemia in Working Women

**DOI:** 10.7759/cureus.44104

**Published:** 2023-08-25

**Authors:** Shahtaj A Shah, Umar Soomro, Ovais Ali, Yumna Tariq, Madeeha Subhan Waleed, Prathima Guntipalli, Nadia Younus

**Affiliations:** 1 Department of Medicine, Jinnah Medical and Dental College, Karachi, PAK; 2 Department of Internal Medicine, Lower Bucks Hospital, Bristol, USA; 3 Department of Internal Medicine, Methodist Health System, Dallas, USA

**Keywords:** iron deficiency anemia (ida), hemoglobin, working women, anemia, prevalence

## Abstract

Introduction: Anemia can be defined as a reduction in the amount of hemoglobin (Hb) in red blood cells (RBCs). It is becoming a growing socioeconomic issue. It is important to identify the causes of anemia and educate people about its symptoms. This can aid in the early identification and diagnosis of anemia, thereby preventing the disease’s complications. The complications of anemia include the risk of low birth weight, prematurity, prenatal and neonatal mortality, and maternal mortality.

Objective: The objective of the study is to investigate the factors contributing to anemia among working-class women employed in government or private sectors located in Karachi, Pakistan. By identifying the causes and risk factors of anemia, participants can be counseled to adopt a healthier lifestyle, a well-balanced diet, and activities that may eliminate the causes of anemia, further preventing the incidence of anemia. The objectives of the study are specific, measurable, achievable, realistic, and time-bound (SMART). The study was conducted from November 2019 to August 2021 despite facing COVID-19-related restrictions. The sample size fit the inclusion criteria, and the objectives were accomplished successfully with adequate resources.

Methodology: The cross-sectional study was conducted after receiving clearance from institutions and consent from participants. A total of 180 participants provided consent after receiving a thorough explanation of the study, and they had the right to refuse to participate. In respect of confidentiality, the participants were not required to provide their names, as they were not included in our data analysis. The inclusion criteria included women of the working class, aged 18-45 years, working a minimum of four to five hours per week, and employed in government or private sectors located in Karachi, Pakistan. The exclusion criteria included women with bleeding or hematological disorders, a history of surgery in the last 12 months, pregnancy, or systemic disease. Data collection was divided into two sections: section one (questionnaire) and section two (investigations). The questionnaire was given to each participant via Google Forms and was filled out before section two of data collection, which included blood tests via a finger prick to measure hemoglobin with a Veri-Q Multi Meter hemoglobin monitoring system (manufactured by Q-line BIOTECH, New Delhi, India).

Results: The mean Hb was 11.15 ± 1.29 mg/dl (n = 180). The study revealed that 58.3% of the participants had a normal hemoglobin concentration according to the WHO anemia classification, while 41.7% were anemic. Overall, the prevalence of anemia was 41.7%, and the majority (56%) of the participants had mild anemia.

Conclusion: The majority of the participants had mild anemia. Education on anemia and its symptoms, eating habits, occupational status, and stress-related factors can affect the hemoglobin concentration in RBCs. A diet low in meat, leafy vegetables, and fruit leads to anemia. Despite knowledge of anemia and its symptoms, non-medical professionals, especially young adults, had a higher prevalence of anemia than medical professionals, although the difference was minor.

## Introduction

According to the World Health Organization (WHO), anemia affects 1.62 billion people worldwide, corresponding to 24.8% of the population. Anemia can be defined as a reduction in the number of red blood cells (RBCs), hemoglobin, or iron content. This results in the abnormal RBC's inability to carry oxygen adequately to tissues in the body. According to complete blood count (CBC) reports, the RBCs can be classified as normocytic (mean corpuscular volume (MCV) 80-100 FL), microcytic (MCV <80 FL), or macrocytic (MCV >100 FL). They are also differentiated according to colors (hypochromic (pale), normochromic (normal/pink to red), or hyperchromic (red)) [1].

Iron deficiency is one of the most common causes of anemia worldwide, with approximately 1.24 billion cases [2]. Blood loss (e.g., menstruation), parasitic infections, thalassemia, and sickle cell disease are also common causes. Many factors, including age, sex, residential elevation (altitude), smoking habits, and pregnancy, affect hemoglobin concentration [3]. Anemia may affect any age group; however, female medical students specifically have been found to have a particular susceptibility to the development of anemia due to their diligent schedules, variable meal times, and long working hours [4]. It is becoming a growing socioeconomic issue as it decreases physical and cognitive productivity [4]. The consequences of anemia include an increased risk of low birth weight babies, premature births, prenatal, neonatal, and maternal mortality [5]. Decreased mental concentration and productivity have also been reported [4]. Therefore, it is important to know the status of iron deficiency anemia, as the productivity of workers depends on their health condition [6]. It is yet to be determined why anemia is more common among working women; however, multiple factors such as stress, workload, financial issues, work environment, and occupation may also contribute to this [7]. A study focused on the prevalence of anemia in premenopausal working women assessed their iron status and found those menstruating or recently pregnant were at a higher risk for iron deficiency [7]. Interestingly, overweight or obese women were found to have a lower risk of the development of anemia in a Chinese population of women, possibly due to an increased intake of iron in obese women [8]. Another study reported that 40% of an African population of working women is affected by anemia, as well as a higher frequency of anemia among women with informal education [9]. A common finding in studies revealed that young working women were more knowledgeable of anemia, its causes, and its consequences than those who were uneducated and residing mostly in villages [10]. Overall, anemia is a condition that many women suffer from worldwide and is more likely to have a higher prevalence in the working class than in non-working women.

Purpose

Conducting our study helped create awareness and educate our participants employed in different government or private sectors located in Karachi, Pakistan, by providing information about anemia, its symptoms, and complications. The diagnosis of anemia based on the hemoglobinometer measurements was provided to those unaware of the presence of their condition. This helped women understand the condition and the importance of prevention, and it motivated many to reduce known potential causes of anemia. This also provided an opportunity to prevent potentially serious complications later in life. As a public health issue, it is the duty of health organizations to take action against the prevalence of anemia and spread public knowledge about the causes, preventions, and consequences of anemia. Hence, our research aimed to evaluate and estimate the prevalence of anemia in the working women's population.

Rationale

The rationale of the study was to investigate anemia's effect on the quality of a woman's life. A healthy woman is fundamental to ensuring the health of future generations. Anemia is a factor that may not only compromise a woman’s life but also poses a risk to potential future pregnancies. Assessing the prevalence of anemia in working women allows us to advise them towards the adoption of a healthier lifestyle, a well-balanced diet, and activities that may eliminate the causes, thereby preventing the occurrence of anemia. It is very important for women to know about this condition because it is a problem that is commonly ignored or not completely understood and has the potential to lead to serious consequences if left untreated.

## Materials and methods

The study aimed to determine the prevalence of anemia in working women by measuring hemoglobin levels with the Veri-Q Multi Meter hemoglobin monitoring system. A total of 180 samples were collected, and a diet-related questionnaire was developed for participants to fill out. The study was conducted in a limited timeframe and was completed by the end of 2020. The study was structured based on a cross-sectional design that was implemented on a sample population of women who worked in the government or private sectors in Karachi, Pakistan. Ethical clearance was obtained from the Undergraduate Research Committee of Jinnah Medical College Hospital in Karachi. Each participant provided written consent after being presented with a form that outlined the study's purpose and procedures. Participants were informed that they had the right to refuse participation. The inclusion criteria for the study were working women between the ages of 18 and 45, while the exclusion criteria were women with bleeding or hematological disorders, a history of surgery in the last 12 months, pregnancy, or systemic disease. The sample size of 180 participants was calculated using a specific formula based on the design effect, population size, and estimated proportion. The formula ensures that the sample size is sufficient to provide values with the desired level of precision and confidence, considering the characteristics of the population and the study design.

The formula used for calculation was [DEFF*Np(1-p)]/ [(d2/Z21-α/2*(N-1)+p*(1-p)] (20)

(n=sample size, deff=design effect, N=population size, pˆ=the estimated proportion, qˆ=1 = pˆ, d=desired absolute precision or absolute level of precision).

A comprehensive, self-administered questionnaire was distributed to collect background information and the socio-demographic status of the participants. The questionnaire also helped gather information regarding the level of education, marital status, dietary habits (intake of fruits, vegetables, and meat), exercise habits, menstrual cycle patterns, history of comorbid conditions, number of visits to the doctor, recurrence of infections, and stress-related questions of the participants. The questionnaire was developed by dividing the questions by topic. The topics included education on anemia, diet, occupational, and stress-related factors. The education topic assessed the general knowledge of the participant regarding health and anemia to develop conclusions based on the collected data. Diet-related questions helped build connections between suspected anemic subjects and their dietary habits. Occupational and stress-related factors were added to understand the participants' daily lives and possible external factors that could contribute to anemia. A finger prick method was used to take the blood sample and analyze it using a Veri-Q Multi hemoglobin monitoring system (manufactured by Q-line BIOTECH, New Delhi, India). IBM Statistical Package for the Social Sciences (SPSS) Version 23 (Armonk, NY: IBM Corp.) was used for statistical calculations.

Anemia is classified by the WHO criteria based on the concentrations of hemoglobin for the diagnosis and assessment of severity. This classification was used to classify anemia in participants according to their hemoglobin concentrations. The classification is shown in Table 1.

**Table 1:** WHO classification of anemia

Severity of anemia	Non-pregnant (15 years and above)
Non-anemic	12.0 mg/dL
Mild anemia	11.0-11.9 mg/dL
Moderate anemia	8.0-10.9 mg/dL
Severe anemia	<8.0 mg/dL

## Results

The mean Hb was found to be 11.15 ± 1.29 mg/dl (n=180); 58.3% (n=105) were normal, while 41.7% (n=75) were anemic, as shown in Table 2.

**Table 2:** The mean hemoglobin (Hb) values of the study participants

Hemoglobin (mg/dl)	Frequency (n)	Percentage (%)	Mean hemoglobin (mg/dl) (mean ± SD)
Normal ≥ 12 mg/dl	105	58.3%	12.03 ± 0.68
Anemia ≤ 11.9 mg/dl	75	41.7%	9.92 ± 0.86

Table 3 categorizes three different age groups and shows the comparison between Hb and age.

**Table 3:** Frequency and comparison of Hb with age

Age (years)	Anemia ≤ 11.9 mg/dl	Normal ≥ 12 mg/dl	Frequency (n)	Percentage (%)	p-value
18-25	43	58	101	56.1%	0.58
26-35	21	25	46	25.6%	
36-45	11	22	33	18.3%	

Among the age group of 18-25 years (56.1%, n=101), 42.5% (n=43) were found to be anemic. Whereas, among the participants of the second age group, aged 26-35 years (25.6%, n=46), 45.6% (n=21) were anemic, while in the age group of 36-45 years (18.3%, n=33), 33.3% (n=11) were found to be anemic.

Another classification, as displayed in Table 4, compared Hb concentration with occupation.

**Table 4:** Frequency and comparison of Hb with the occupation of the study group

Occupation	Anemia ≤ 11.9 mg/dl	Normal ≥ 12 mg/dl	Frequency (n)	Percentage (%)	p-value
Medical	63	90	153	85%	0.751
Non-medical	12	15	27	15%	

As per the table, 85% (n=153) of the participants were medical professionals, while the remaining 15% (n=27) belonged to non-medical professions. Out of the medical professionals, 41.1% (n=63) were found to be anemic, while out of the remaining non-medical professionals, 44.4% (n=12) were found to be anemic.

The participants' understanding and knowledge of anemia were assessed through questionnaires provided by the investigators. The questionnaire revealed that 96.1% of participants correctly identified that men are not considered a high-risk group for anemia, while only 3.9% indicated that men are a high-risk group. This suggests that the participants possess a general understanding that women, regardless of age or profession, are more susceptible to anemia. The questionnaire also assessed the understanding of the symptoms of anemia by the study participants. As displayed in Table 5, only 38.9% (n=70) of the participants correctly identified pale gums as being a symptom of anemia, while 61.1% (n=110) stated no association between pale gums and anemia. However, 62.8% (n=113) of the participants correctly identified the presence of nail appearance changes in anemia, while 37.2% (n=67) responded that there was no known association.

**Table 5:** Frequency and comparison of Hb with the participants' knowledge of anemia

Symptom	Response	Anemia ≤ 11.9 mg/dl	Normal ≥ 12 mg/dl	Frequency (n)	Percentage (%)	p-value
Pale gums	No	48	62	110	61.1%	0.50
	Yes	27	43	70	38.9%	
Dark circles under the eyes	No	60	67	127	70.6%	0.01
	Yes	15	38	53	29.4%	
Numbness of hands and feet	No	41	63	104	57.8%	0.47
	Yes	34	42	76	42.2%	
Changes in the appearance of nails	No	30	37	67	37.2%	0.51
	Yes	45	68	113	62.8%	

When comparing Hb with the eating habits of the participants, 36.6% (n=66) of the participants consumed less than two meals per day, out of which 46.9% (n=31) were anemic. Out of the 63.3% (n=114) who consumed more than two meals per day, only 38.5% (n=44) were found to be anemic. The comparison of Hb with eating habits was further assessed, as displayed in Table 6.

**Table 6:** Frequency and comparison of Hb with the participants' eating habits

Food intake	Response	Anemia ≤ 11.9 mg/dl	Normal ≥ 12 mg/dl	Frequency (n)	Percentage (%)	p-value
Meat	≤ 2/week	51	60	111	61.6%	0.12
	≥ 3/ week	24	45	69	38.3%	
Leafy vegetables	≤ 2/week	42	59	101	56.1%	0.66
	≥ 3/ week	33	46	79	43.9%	
Fruits	≤ 2/week	48	62	110	61.1%	0.62
	≥ 3/ week	27	43	70	38.9%	

The results revealed that out of the 61.6% (n=111) of the participants who consumed meat two times or less per week, 45.9% (n=51) of them were anemic, while in the group of 38.3% (n=69) who consumed meat three or more times per week, 34.7% (n=24) were found to be anemic. Similarly, 56.1% (n=101) of the participants consumed leafy vegetables twice or less per week, out of which 41.5% (n=42) were found to be anemic. Among 43.9% (n=79) of the participants who consumed leafy vegetables three or more times per week, 41.7% (n=33) were found to be anemic. The results for the inclusion of leafy vegetables in the diet showed similar results, regardless of consumption. In addition, fruit consumption was assessed; out of the 61.1% (n=110) who reported consumption of fruits twice or less per week, 43.6% (n=48) were found to be anemic, while in the group of 38.9% (n=70) of those who consumed fruits three or more times per week, 38.5% (n=27) were anemic.

**Figure 1:**
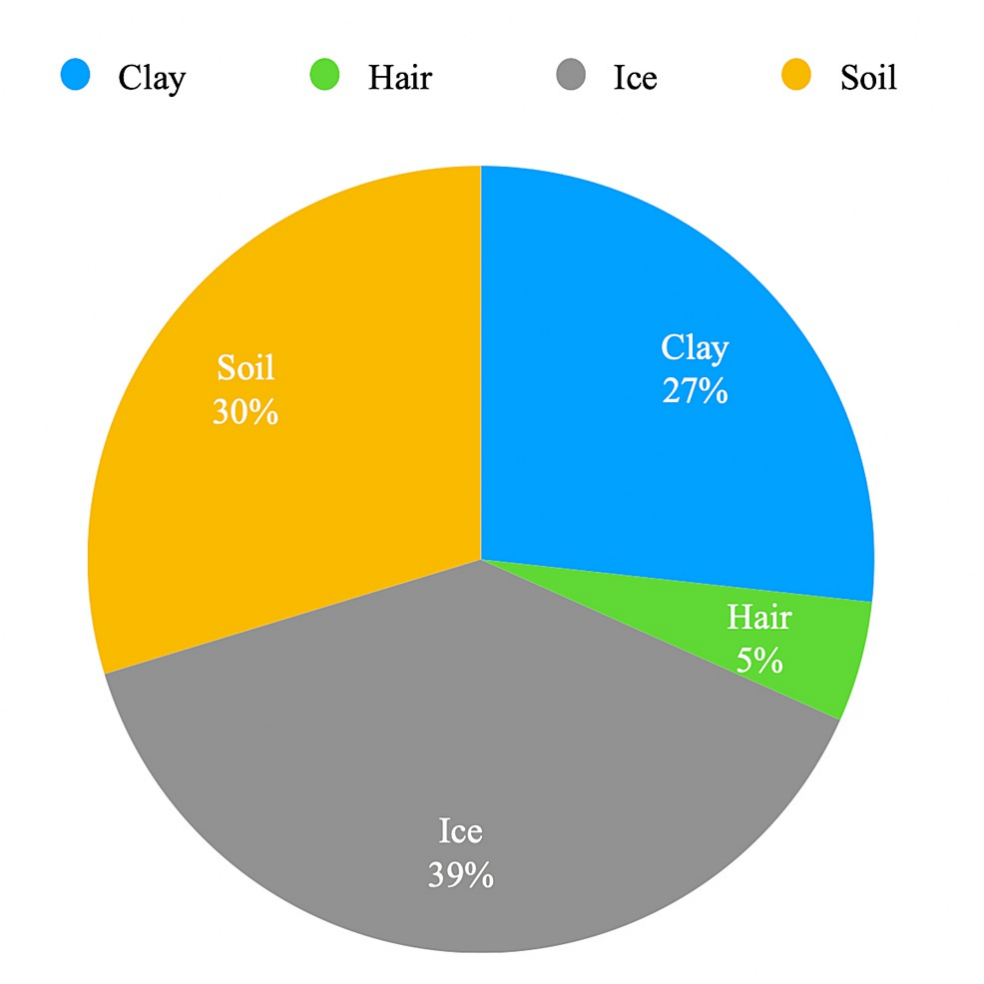
Common cravings of participants who reported having pica

In addition, Hb concentration and the presence of pica were assessed; 58.9% (n=106) did not report any symptoms of pica, whereas 41.1% (n=74) reported having pica. Amongst those who reported the presence of pica, ice was the most common craving (39%), followed by soil (30%), clay (27%), and hair (5%) (Figure 1).

Of the 58.9% who reported no pica, 42.4% were anemic, while of the 41.1% with pica, 40.5% were anemic.

Lastly, the majority of the known comorbidities reported by participants in our study included asthma and hypertension, respectively. Diabetes and thyroid conditions were also reported, although less commonly. Overall, the prevalence of anemia was 41.7%, and among this, the majority (56%) of the participants had mild anemia.

## Discussion

The prevalence of anemia varies widely according to factors such as age, gender, physiologic causes, pathologic causes, nutritional factors, and socioeconomic conditions. In our study, we found a significant correlation between anemia and working women. Different studies have shown a significant relationship between being underweight and anemia. Bano et al. stated that the majority (81.8%) of anemic students were undernourished according to their BMI, whereas Pandey et al. stated that the prevalence of anemia among underweight students was 60%, normal was 27.5%, and overweight was 12.5% [11, 12]. In our study, 101 (56.1%) consumed green leafy vegetables two times or less per week, out of which 41.5% were found to be anemic, while for those who consumed three or more in a week, 41.7% were anemic. Hadaye et al. reported similar decreased compliance toward healthy eating in their study at a tertiary care hospital in Mumbai, India, in which 70 out of 280 students (25%) consumed green leafy vegetables only once a week [13]. Although the diagnosis of anemia is simple, it goes unnoticed for a prolonged duration due to its non-specific clinical signs and a lack of testing and patient education on the symptoms of anemia. As anemia is associated with a low work capacity as well as lasting effects on learning and cognitive function, attention, behavior, and growth, identification of the disease and contributing factors is a first step toward its management [14].

Anemia is a very common health and economic burden. Anemia is a preventable and treatable condition if local factors are recognized in time. According to the World Health Organization, the prevalence of anemia is even greater than 40% [15]. Hence, it is a global problem of great magnitude. In our study, the prevalence of anemia in the overall population was 41%. In non-pregnant women between 15 and 49 years of age, the worldwide prevalence of anemia has declined from 31% to 30% from the year 2000 to 2019, whereas for pregnant women in similar age groups, it has decreased from 41% to 36% [16]. Our study showed an incidence of anemia in 42.5% of women aged 18-25, 45.6% of women aged 26-35, and 33.3% of women aged 36-45. In the year 2019, the incidence of anemia in women of the reproductive age group was 30.1% [17], with iron deficiency being the leading cause of this condition [18]. The prevalence of iron deficiency anemia (IDA) in younger women is 2.4%, as compared to 5.5% in older women [16].

Education is a common predictor of the risk of anemia. A study conducted among educated Thai women showed 21% had anemia, of which 85% were cases of IDA [19]. Factors that can lead to anemia in women include increased age, lack of education or awareness, high parity, low socioeconomic status, and poor nutritional status. Improving literacy and diet will improve anemia, especially in developing countries [20]. Our article compares anemia in women from a medical profession compared to a nonmedical profession. The incidence was 41.1% in women belonging to the medical profession versus 44.4% in women belonging to the non-medical profession. Increasing awareness about the condition and improving access to fortified foods can help reduce the incidence of anemia [21]. Another study assesses anemia in women working in the textile industry, with iron deficiency anemia (IDA) being a major cause of anemia in women [15].

Pica is a symptom that occurs commonly with iron deficiency. Pregnant women and iron-deficient individuals are the most susceptible. Iron therapy usually cures pica [22]. In our study group, 41.1% reported having pica. The most common craving was ice. A double-blind study concluded that iron-deficient individuals had a craving for ice, and the craving improved with iron supplementation [23]. However, this association has not been proven. Other symptoms associated with iron deficiency anemia include restless leg syndrome, decreased concentration, fatigue, and cardiovascular stress.

Anemia is related to a variety of comorbid conditions; IDA is more common in diabetic individuals and can predispose diabetics to more complications [24]. Consequently, diabetes itself is a cause of chronic kidney disease and end-stage renal disease (ESRD), which also causes renal anemia. Ten percent of the participants had diabetes mellitus. Hypertensive individuals with anemia have poor outcomes. Hypertensive individuals with anemia are at increased risk of cardiovascular and renal events as compared to hypertensive individuals without anemia [25]. Many of our individuals with anemia had comorbid hypertension. Normocytic anemia is highly prevalent in hypertensive patients [26]. Patients with asthma are more prone to anemia as well; the prevalence in patients with asthma is 1.25 times higher than in individuals without asthma [27]. The most prevalent comorbid condition with anemia described in our research was asthma. Although the global prevalence of anemia has decreased over time, its burden remains high. According to our study, the prevalence of anemia in working women was 41.7%, with a mean Hb value of 11.15 ± 1.29 mg/dl (mean ± SD). As the scope of our study was limited, further research may be beneficial for a better understanding of anemia, especially in adolescent populations.

Limitations

Certain limitations were faced, including the limited amount of research and exposure to the public sector. The constraints of finding non-medical professionals for the research were due to COVID restrictions, lockdowns, and permissions, which also contributed to the limitations of the study. There was also reluctance among many participants when asked to give a blood sample.

## Conclusions

The prevalence of anemia in working women in this study was 41.7%, and the majority of our participants had mild anemia. Education on anemia and its symptoms, eating habits, occupational status, and stress-related factors can affect hemoglobin concentration in RBCs. Factors including a diet low in meat, leafy vegetables, and fruit lead to anemia. Despite knowledge of the condition and its symptoms, non-medical professionals, especially young adults, had a higher prevalence of anemia as compared to medical professionals, although the difference was minor. Although the global prevalence of anemia has decreased over time, its burden remains high. More research should be conducted annually, specifically for women in reproductive age groups, to assess the prevalence of anemia and the causative factors of a higher prevalence, whether due to a lack of education about anemia, work-related factors, or malnourishment. Including an equal number of working and non-working participants might be beneficial for a stronger comparison. Frequent studies will allow the chance to intervene and educate the specific high-risk age groups to decrease the frequency of anemia amongst working women.
